# Nutritional Content Dynamics and Correlation of Bacterial Communities and Metabolites in Fermented Pickled Radishes Supplemented With Wheat Bran

**DOI:** 10.3389/fnut.2022.840641

**Published:** 2022-03-08

**Authors:** Xiaoqiong Li, Daqun Liu

**Affiliations:** Food Science Institute, Zhejiang Academy of Agricultural Sciences, Hangzhou, China

**Keywords:** wheat bran, bacterial community, correlation analysis, nutrients, flavor, fermented pickled radish

## Abstract

Wheat bran supplementation in cereal food processing improves the nutritional value and quality of the final products. However, whether wheat bran has the potential as a biofortifier to enhance nutritional and flavor of fermented vegetables remains unknown. The study aimed to evaluate the potential of wheat bran supplementation for nutrition and flavor fortification during radish fermentation, and to explore the role of microorganisms in nutritional and flavor development. Using high-throughput sequencing coupled with high-performance liquid chromatography and headspace solid-phase microextraction-gas chromatography-mass spectrometry, the microbial community profiles and nutritional and flavor changes of wheat bran-treated samples were analyzed and compared with control samples. Correlation analysis between bacteria taxa with metabolites were also performed. The results showed that wheat bran treatment increased the content of most free amino acids (FAAs), α-linolenate, thiamine, and riboflavin in the samples (*p* < 0.05). In addition, the increased consumption of reducing sugar and glutamate in the wheat bran-treated samples was due to the production of secondary metabolites such as lactic acid, ethanol, acetic acid, and GABA (*p* < 0.05). Moreover, compared with control samples, the flavor of the wheat bran-treated pickled radish was preferable. Wheat bran increased the amount of alcohol, ester, acid, and ketones compounds but reduced the number of sulfides, which increased the aroma but decreased the pungent flavor. Additionally, the correlation analysis suggested that *Lactobacillus*, the most dominant genus, was boosted by wheat bran and was positively associated with most of FAAs, GABA, and lactate, while negatively associated with most sulfides. Therefore, compared with the control, wheat bran treatment could improve the nutritional values and sensorial properties of radish pickles. New areas of research should explore the co-fermentation of other vegetables with wheat bran, and the potential of this processing technique to provide consumers with products of high nutritional quality.

## Introduction

*Raphanus sativus* (radish), which belongs to the *Brassicaceae* family, has attracted attention in the scientific community due to its valuable source of nutritional and pharmaceutical compounds, particularly dietary fiber, proteins, glucosinolates, flavonoids, β-carotene, and minerals (1, 2). Radish roots can also be fermented and are most commonly consumed in Asia (3–5). During fermentation, unique flavors and metabolites are produced, which promote the taste, aroma, and texture of pickled radishes (6). Glucosinolates are sulfur-containing nonvolatile flavor precursors that are enzymatically hydrolyzed into volatile flavors when the cellular structure is disrupted during fermentation. Initial products include isothiocyanates, while further reactions may form thiols, sulfides, disulfides, and trisulfides, which are responsible for the off-odor characteristics of pickled radishes (7). Furthermore, various sulfur-containing products, alcohols, acetic acid, carbonyl, and acetal compounds dominate the volatile fractions of pickled radishes (6, 8).

Despite modern technology to efficiently batch produce fermented foods on an industrial scale, traditional fermentation methods are still widely used and preferred in small-scale factories or at home (6). However, there are geographical differences in the radish pickling process. In the Sichuan province, the process for radish pickling is relatively simple, where the fresh radish roots are first pretreated and soaked in aged brine containing 6% NaCl with a pH of 3.5, where they undergo spontaneous fermentation at room temperature (6). In the Zhejiang province, spontaneous or natural fermentation consists of three stages, where the radish roots are first dehydrated at high salt concentrations of 6–8% (w/w) for ~1 week. After draining the brine, the radish roots are dehydrated for another week at low salt concentrations of 2–3% (w/w). Then the dehydrated radish roots are further fermented with condiments for desalting and seasoning. Throughout the fermentation process, the radish roots are compressed with weight stones and kept in a cool and well-ventilated environment (9). Besides, in Japan, *Takuan-zuke*, fermented Japanese winter radish root (daikon) consists of salt, rice bran (*Nuka*), and daikon (10). The addition of rice bran to *Takuan-zuke* during the salt-aging process increases niacin, glutamate, and acetate production (4). Consequently, geographical and production process differences may result in unique flavors and the nutritional value of the fermented pickled radishes. Thus, in this study, we used wheat bran instead of rice bran, and evaluated its impact on the nutrition and flavor of pickled radish root.

The formation of functional nutrients and flavor compounds in fermented food is comprehensively affected by the processing conditions, endogenous enzymes, microorganisms, seasonings, and additives. Among these, microorganisms, particularly lactic acid bacteria (LAB), play a key role in the formation of unique flavors (4, 6, 10–12). For example, LAB produce lactic acid, which suppresses the growth of bacteria and microorganisms that can cause food poisoning and spoilage; therefore, LAB can improve the safety, quality, and shelf life of pickled radishes (13, 14). Meanwhile, LAB may form secondary metabolites, which can affect the flavor and chemical characteristics of fermented products (15). For instance, some *lactobacilli* species are facultative hetero-fermentative and can transform glucose and lactose into lactic acid and subsequently into acetic acid while producing other metabolites with desirable properties (16). Furthermore, LAB are generally recognized as safe (GRAS) and can produce bacteriocins, exopolysaccharides, or bioactive substances, such as γ-aminobutyrate (GABA), which offer nutritional and health benefits (17, 18). Thus, by analyzing the relationship between microorganisms and flavor compounds we can identify potential flavor-forming microorganisms, and by modifying the flora we can regulate the flavor characteristics of fermented foods.

In this study, we sought to monitor the nutritional value changes during the entire fermentation process, including during the initial, maturation, and deterioration stages. In addition, we investigated the effects of adding wheat bran on the nutritional value, flavor, and bacterial community of ripe pickled radishes at the mature stage. Moreover, we revealed the correlation between bacterial community and the metabolites of ripe pickled radishes. The results of our study provide a greater understanding of the fermentation mechanisms of fermented radishes and help to determine whether wheat bran supplementation can improve the nutrition and taste of pickled radishes.

## Materials and Methods

### Radish Fermentation and Sample Collection

We obtained a total of 300 kg of fresh white radish roots from a local supermarket in Hangzhou, China. The radish roots were dehydrated and fermented according to the methods described by Liu et al. (19), with minor modifications (Supplementary Figure S1). During the dehydration stage, radish roots in six containers were dehydrated at 6%, and then 2% (w/w) salt concentrations for 1 week each, and the brine was drained weekly. Radish root samples were sterilely collected from three containers on days 0 and 14 and labeled as the dehydration group (DEHY, *n* = 3). On the 15th day, the pickled radish roots were transferred to new marinating containers and divided into two groups. For the control groups (CONT, *n* = 3), the salted radish roots were mixed with a seasoning mixture containing sugar, concentrated aspartame (100×), citric acid, and sodium glutamate at 6.29, 0.0629, 0.2, and 1.2% (w/w/w/w), respectively. For the treatment groups (BRAN, *n* = 3), the salted radish roots were mixed with 5% of wheat bran (5/100 g), in addition to the seasoning mixture. Throughout the fermentation process, the radish roots were compacted and maintained in a cool (18–20°C) and well-ventilated environment. Sterile sampling was conducted randomly, where the radish root samples were taken from each container after 28, 42, and 56 days, to determine their nutritional content. Physicochemical and organoleptic indicators suggested that the pickled radishes were ripe on the 28th day. Therefore, samples on the 28th day forward were characterized or showed volatile flavor profile and bacterial community.

### Amino Acid Analysis

#### Free Amino Acid

We analyzed the content of 16 different free amino acids (FFAs) [aspartic acid (Asp), glutamic acid (Glu), serine (Ser), glycine (Gly), threonine (Thr), alanine (Ala), proline (Pro), lysine (Lys), histidine (His), arginine (Arg), tyrosine (Tyr), valine (Val), methionine (Met), isoleucine (Ile), leucine (Leu), and phenylalanine (Phe)] in the fermented radish samples *via* high-performance liquid chromatography (HPLC) with precolumn derivatization using phenylisothiocyanate, according to the GB 5009.124-2016 standard (National Standard of China), and the test was conducted at the Zhejiang Impartial Inspection Center Co., Ltd. (Hangzhou, China). First, 0.5 g of each sample was digested with 6 mol/L of HCl at a constant temperature of 110°C for 22 h. After the samples were filtered and re-dissolved, the resulting solution was injected into an HP1100 amino acid analyzer (Agilent, USA), equipped with an ion exchange column, and the amino acid content was obtained according to the standard references (20). The results were expressed as mg/100 g of sample.

#### γ-aminobutyrate (GABA)

The GABA content in the fermented radish samples was assessed according to the QB/T 4587-2013 Chinese industry standard. Approximately 1.0 g of pickled radishes were ground in 5 mL of 2% sulfosalicylic amino acid at 4°C, and the pH of the homogenate was maintained at 2.0 by adding 0.02 M HCl. Then the samples were centrifuged at 10,000 g at 4°C for 15 min. The supernatant was used to determine the GABA content via an HP1100 amino acid analyzer (Agilent, USA) (21).

### Organic Acid Analysis

The contents of four organic acids, citrate, malate, lactate, and fumarate, were determined using HPLC according to the Chinese national standard (GB 5009.157–2016). The samples were handled according to the methods described in Yang et al. (22), with slight modifications. Separation was performed on an Amethyst C18–H column (5 μm particle size, 4.6 × 250 mm, Sepax Technologies, Inc., Delaware, USA) at 30°C using CH_3_OH/H_2_O (3:97, v/v) as the eluent, and at a flow rate of 0.6 mL/min. Quantification was conducted using an external standard method, and the organic acid content results were expressed as mg/g or μg/g.

### Fatty Acid Analysis

The content of four fatty acids, palmitate, stearate, oleate, and α-linolenate, in the fermented radish samples was determined according to the GB 5009.168-2016 Chinese National Standard. Trichloromethane and methanol solution (3 mL) was added to 0.5 g of sample until sufficiently homogenized. After filtering the residue, 4 mL of deionized water was added, and the samples were centrifuged at 3,000 rpm. The organic solvent retained from the oily layer was removed using a water bath at 40°C for 12 h, after which 1 mL of n-hexane was added to the samples and homogenized. Then, potassium hydroxide (0.4 M) and 1 mL of methanol solution were added, and the samples were shaken for 1 h. After 30 min, 2 mL of deionized water was added, and the samples were centrifuged (3,000 rpm). Then, the supernatant was used for GC analysis according to the methods described by El-Dakar et al. (23). Fatty acid content was determined by referring to the standards, and the results were expressed as mg/100 g of sample.

### Sugar Analysis

Fructose, glucose, maltose, and saccharose values in the fermented radish roots were determined according to the GB 5009.8-2016 national food safety standard. This method was previously reported by our group, with slight modifications (24). Radish sample (2.0 g) extraction was conducted by ultrasonic extraction for 30 min by adding 25 mL of water, and then the mixture was passed through 0.22 μm polytetrafluoroethylene filters. The mixture was then analyzed with an Agilent 1,200 HPLC system (Agilent, Palo Alto, CA, USA) with an NH2 column (250 × 4.6 mm, i.d. × 5 μm; Agilent). The available sugars were separated at 30°C using a mobile phase acetonitrile aqueous solution (70%, v/v) with a flow rate of 1.0 mL/min. The compounds were then quantified using external standards and expressed as g/100 g of sample.

### B Vitamin Analysis

Quantitative determination of vitamins B1 (thiamine), B2 (riboflavin), and B3 (niacin) was carried according to the GB 5009.84-2016 and GB 5009.85-2016 Chinese national food safety standards, using an HPLC system (Agilent 1260, Agilent, Palo Alto, CA, USA) equipped with a fluorescence detector, and a ZORBAX SB-Aq C18 column (4.6 × 250 mm, 5 μm). A 0.05 mol/L sodium acetate/methanol solution (65:35) was used as the isocratic solvent system, and the flow rates for thiamine, niacin, and riboflavin were 0.8 and 1.0 mL/min, respectively. In addition, excitation and emission wavelengths for thiamine and niacin were 375 and 435 nm, respectively, and the excitation and emission wavelengths for riboflavin were 462 and 522 nm, respectively (25).

### Volatile Flavor Component Analysis of the Ripe Pickled Radishes

The abundance of volatile compounds was determined using headspace solid-phase microextraction-gas chromatography-mass spectrometry (HS-SPME/GC-MS) according to the methods described by Wang et al. (26). First, 2.0 g of minced radish roots samples on the 28th fermentation day was precisely weighed and transferred into a 10 mL headspace vial containing 5 mL of salt saturated water. Then the headspace (HS) volatiles were extracted by heating the samples to 60°C for 30 min with an SPME fiber (65 μm PDMS/DVB) inserted into the headspace. The SPME fiber was inserted into the injector port of the Agilent 6890N-5975 GC–MS (Agilent, Palo Alto, CA, USA), and desorption was performed within 5 min in splitless mode at 250°C. Volatile metabolite separation was conducted using a VF-waxus capillary column (30 m × 0.25 mm inner diameter, 0.25 μm film thickness, Agilent). The volatile components were then identified by comparing the mass spectra with the NIST MS Search 2.20 database and the reported retention index (RI). The positive and negative matching degrees were >800 (maximum 1,000), and all of the chemical data were normalized according to the internal standard. For volatile quantification, the relative compound content was calculated using the peak area normalization method.

### High-Throughput Sequencing Analysis in the Ripe Pickled Radishes

Radish roots from each fermentation container on the 28th fermentation day were rinsed twice with sterile PBS solution to remove any loosely attached cells (27). Then the central portion of the roots was removed and diced with a sterile scalpel and forceps under sterile conditions. Subsequently, 20.0 g of radish samples was transferred into a Stomacher^®^ bag containing 50 mL of sterile saline solution (0.9% NaCl) and homogenized in a Stomacher^®^ device (Lab Blender, Dawei, Hangzhou, China) for 5 min at room temperature. The resulting radish suspensions were frozen at −80°C before further analysis was conducted. The microbial genomic DNA was extracted from these samples according to the methods described by Zhang et al. (28), using an E.Z.N.A.^®^ Soil DNA Extraction Kit (Omega Bio-tek, Norcross, GA, U.S.).

The 799F/1193R primer pair targeting the V5–V7 region was used to amplify the bacterial 16S rRNA gene and exclude amplification of chloroplastic DNA from the plants. Next-generation sequencing (2 × 300 bp paired-end) was performed using an Illumina MiSeq system at TinyGene Technologies Co., Ltd. (Shanghai, China), according to the methods described by Liu et al. (29). Operational taxonomic units (OTUs) were clustered with 97% similarity using UPARSE (version 7.1), and the chimeric sequences were identified and removed with UCHIME. Taxonomy was assigned using the Silva database v.132 with a confidence threshold of 0.7 and Uclust classifier in QIIME.

### Statistical Analysis

GraphPad Prism software (version 9.0.2) was used to perform statistical analysis. All analyses were conducted with three biological replicates, and data comparison between the two groups was conducted using Student's *t* test (qualitative data, equal variance) or Welch's *t* test (qualitative data, unknown variance). Differences were considered significant when *p* < 0.05. Lastly, correlation networks were calculated between the metabolites and the bacterial taxa using Spearman's rank correlations.

## Results

### Nutritional Profiles

#### Free Amino Acid Profiles

**Figure 1:**
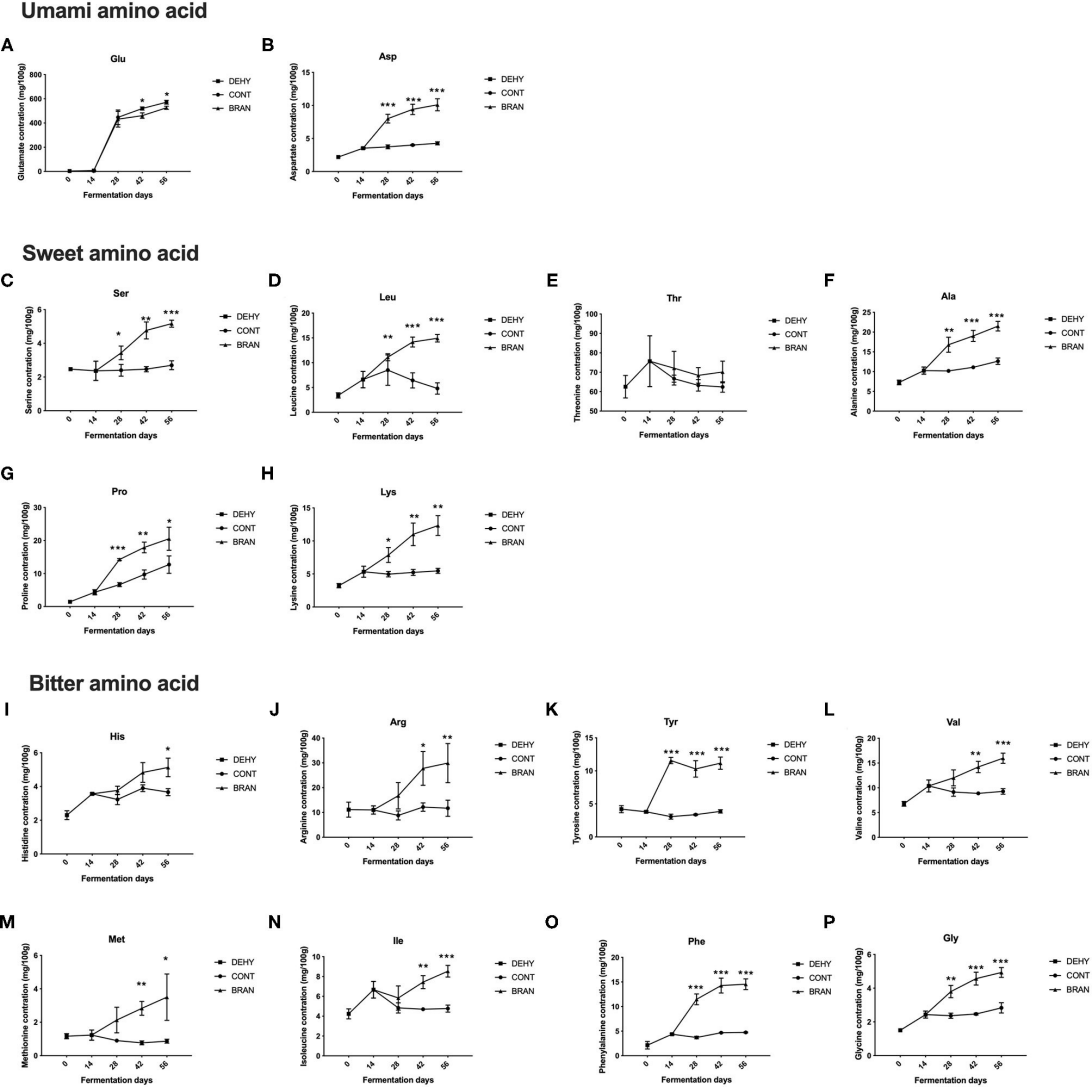
Changes in FFA concentrations during the pickling process of radish roots, showing umami amino acid **(A, B)**, sweet amino acid **(C–H)**, and bitter amino acid **(I–P)**, where DEHY: dehydration stage, including days 0 (the start of dehydration) and 14, CONT: seasoning stage (days 28, 42, and 56) of the fermented radish without wheat bran, and BRAN: seasoning stage (days 28, 42, and 56) of the fermented radish with wheat bran. The data are presented as mean ± SD (*n* = 3), and significant differences within each seasoning period between CONT and BRAN are marked as *p < 0.05, ***p < 0.001.

A total of 16 FFAs were tested to assess the effects of wheat bran supplementation on the FFA profiles (Figure 1). As fermentation progressed, most FFA concentrations in the CONT group remained relatively stable compared to the profiles from the initial stage, except for Glu and Pro, which exhibited increased content during the maturation stage. Glutamate was the most abundant FAA in the ripe pickled radishes, followed by threonine, which was the second most abundant.

Unsurprisingly, wheat bran supplementation resulted in significantly increased levels of most FFAs during the maturation and/or deterioration stages (except Glu and Thr). Glutamate was the only FFA that increased less in the BRAN group compared to the CONT group (day 42: 461.3 vs. 519.7 mg/100 g, *p* = 0.018; day 56: 525.9 vs. 571.7 mg/100 g, *p* = 0.016, Figure 1A). The lower Glu content in the BRAN group was attributed to increased GABA pathway metabolism, as evidenced by the increase in Pro, Arg (Figures 1G,J), and GABA production in the BRAN group. By contrast, for umami FFA flavoring, Asp content increased when wheat bran was added, from 3.7 to 8.0 mg/100 g during the ripening stage (day 28) (Figure 1B). Concentrations of sweet-tasting FFA, specifically Ser, Leu, Ala, Pro, and Lys, almost all doubled in the BRAN group compared to the CONT group (Figures 1C,D,F–H). Thus, wheat bran addition resulted in an increase in bitter-tasting FFAs, namely His, Arg, Tyr, Val, Met, Ile, Phe, and Gly (Figures 1I–P).

#### GABA Profiles

**Figure 2:**
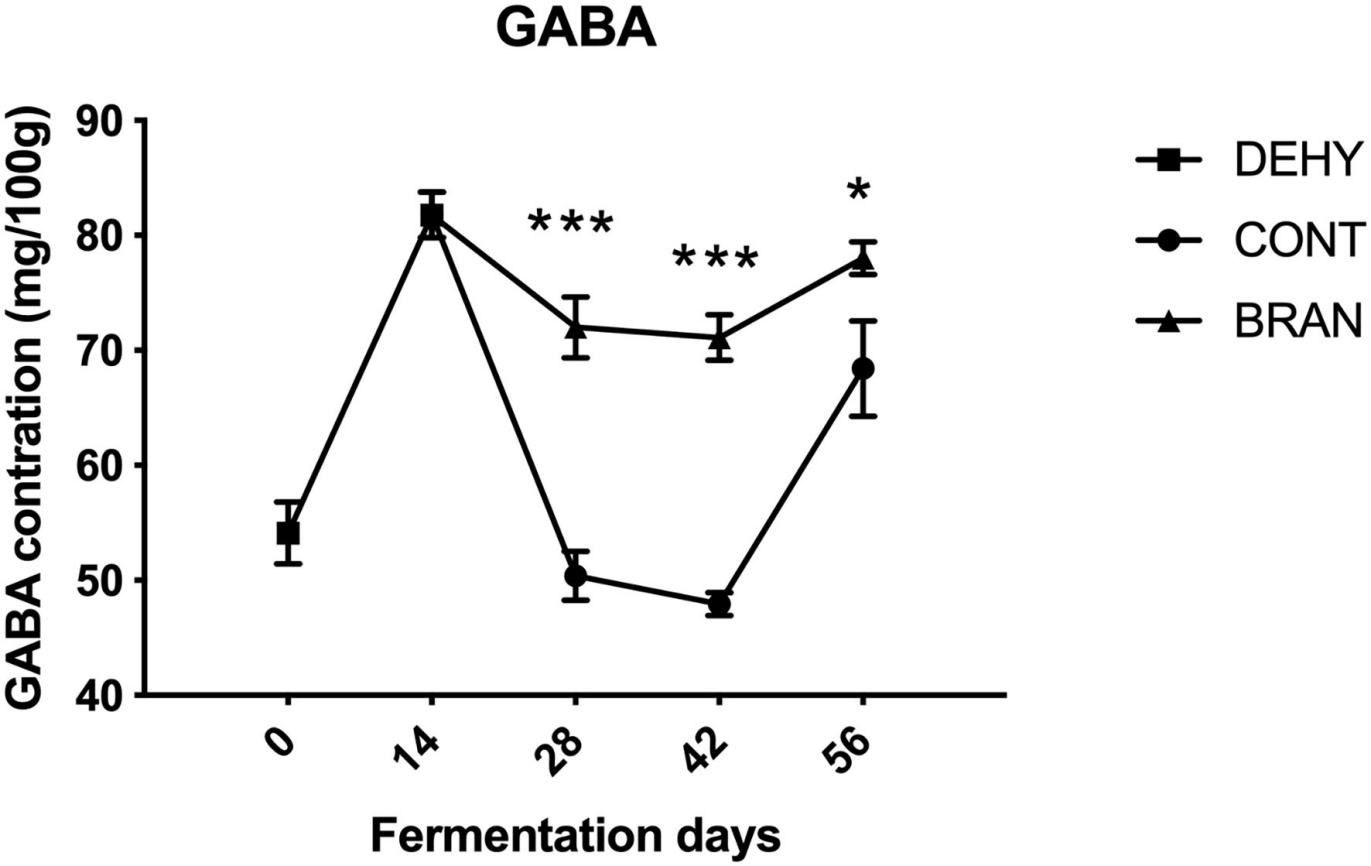
Changes in GABA concentration during pickling of the radish roots. DEHY: dehydration stage, at 0 (start of dehydration) and 14 days, CONT: seasoning stage (days 28, 42, and 56) of the fermented radishes without wheat bran, and BRAN: seasoning stage (days 28, 42, and 56) of fermented radishes with wheat bran. The data are presented as mean ± SD (n = 3), and significant differences within each seasoning period between CONT and BRAN are marked as *p < 0.05, ***p < 0.001.

Changes in GABA content during radish root fermentation were compared with and without wheat bran (Figure 2). First, dehydration resulted in increased GABA content after 14 days, compared to the fresh radish roots (81.8 vs 54.1 mg/100 g). However, in the CONT group, GABA levels dropped to 47.9/100 mg/g during the maturation stage, then increased to 68.4/100 mg/g during the deterioration stage. Thus, the wheat bran prevented GABA level from sharply dropping, causing the BRAN group to exhibit significantly higher content than the CONT group during the ripening stage (day 28: 72.0 vs 50.4/100 mg/g, *p* < 0.001; day 42: 71.1 vs 47.9/100 mg/g, *p* < 0.001).

#### Organic Acid, Fatty Acid, Sugar, and B Vitamin Profiles

**Figure 3:**
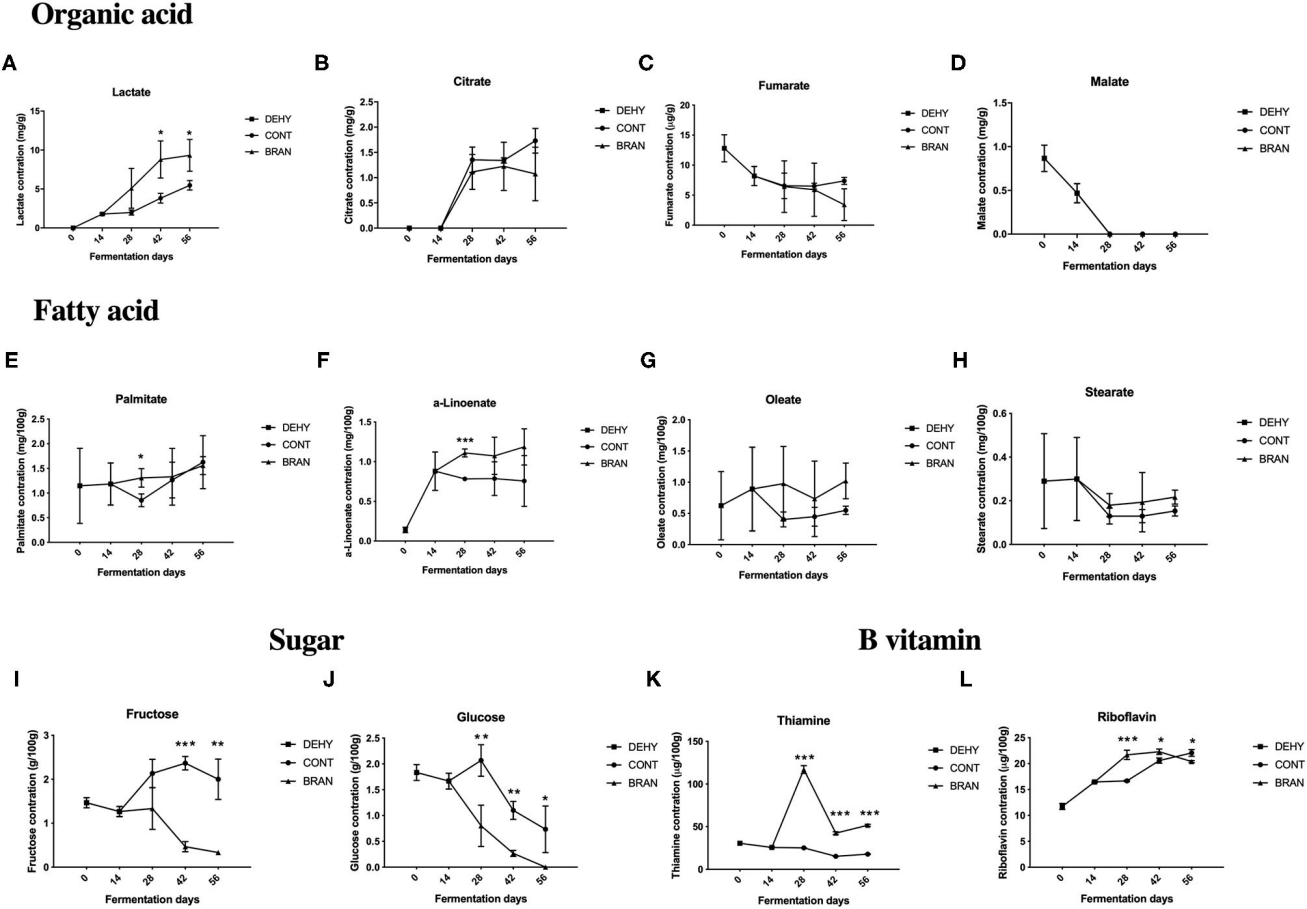
Changes in organic acid **(A–D)**, fatty acid **(E–H)**, sugar **(I, J)**, and B vitamin **(K, L)** concentrations during the pickling process of radish roots. DEHY: dehydration stage, including days 0 (start of dehydration) and 14, CONT: seasoning stage (days 28, 42, and 56) of the fermented radish without wheat bran, BRAN: seasoning stage (days 28, 42, and 56) of the fermented radish with wheat bran. The data are presented as mean ± SD (*n* = 3), and significant differences within each seasoning period between CONT and BRAN are marked as *p < 0.05, **p < 0.01, ***p < 0.001.

Of the four tested organic acids, the lactate and citrate levels increased during the maturation stage after 28 days (Figures 3A,B), whereas the levels of fumarate and malate were decreased by dehydration (Figures 3C,D), and the content of malate was not detected (<0.3 mg/g) as fermentation progressed. Lactate was the major acid product in the fermented radish root, which significantly increased when addition of wheat bran (day 42: 8.8 vs 3.8 mg/g, *p* = 0.025; day 56: 9.3 vs 5.5 mg/g, *p* = 0.035).

Figures 3E–H show the fatty acid concentrations of the fermented radish root samples, including palmitate, α-linolenate, oleate, and stearate. Adding wheat bran only had a significant effect on palmitate content (1.31 vs 0.85 mg/100 g, *P* = 0.026) and α-linolenate (1.11 vs 0.78 mg/100 g, *P* < 0.001) on the 28th day of fermentation, but had no significant effects on the oleate and stearate content throughout the fermentation process.

Among the four tested sugar types (glucose, fructose, maltose, and saccharose), maltose was not detected (< 0.2 g/100 g) throughout all the fermentation stages, and saccharose was only detected in the CONT group after 28 and 42 days of fermentation. Both glucose and fructose were consumed during the fermentation process in the BRAN group (Figures 3I,J). However, glucose and fructose levels initially increased on the 28th day and then decreased during the deterioration stage in the CONT group (day 56). Thus, the addition of wheat bran resulted in a decrease in glucose (day 28: 0.8 vs 2.1g/100 g, *P* = 0.012; D42: 1.1 vs 0.32 g/100 g, *P* = 0.001) and fructose (D42: 0.5 vs 2.4 g/100 g, *P* < 0.001) concentrations in the pickled radishes during the maturation stage.

Among the three tested B vitamins (thiamine, riboflavin, and niacin), niacin was not detected (< 0.03 mg/100 g) throughout all fermentation stages, and in the CONT group, thiamine levels were relatively constant throughout fermentation (Figures 3K,L). However, in the BRAN group, thiamine levels dramatically increased on the 28th day and then decreased on days 42 and 56. Thus, wheat bran treatment increased both thiamine and riboflavin concentrations in the pickled radishes during the maturation stage (day 28: 117.0 vs. 25.2 μg/100 g, *p* < 0.001; day 42: 42.4 vs. 15.3 μg/g, *p* < 0.001). However, on the 56th, riboflavin content in the CONT group surpassed that of the BRAN group (22.1 vs. 20.4 μg/100 g, *p* = 0.013).

### Volatile Comparison Between the CONT and BRAN Groups

**Figure 4:**
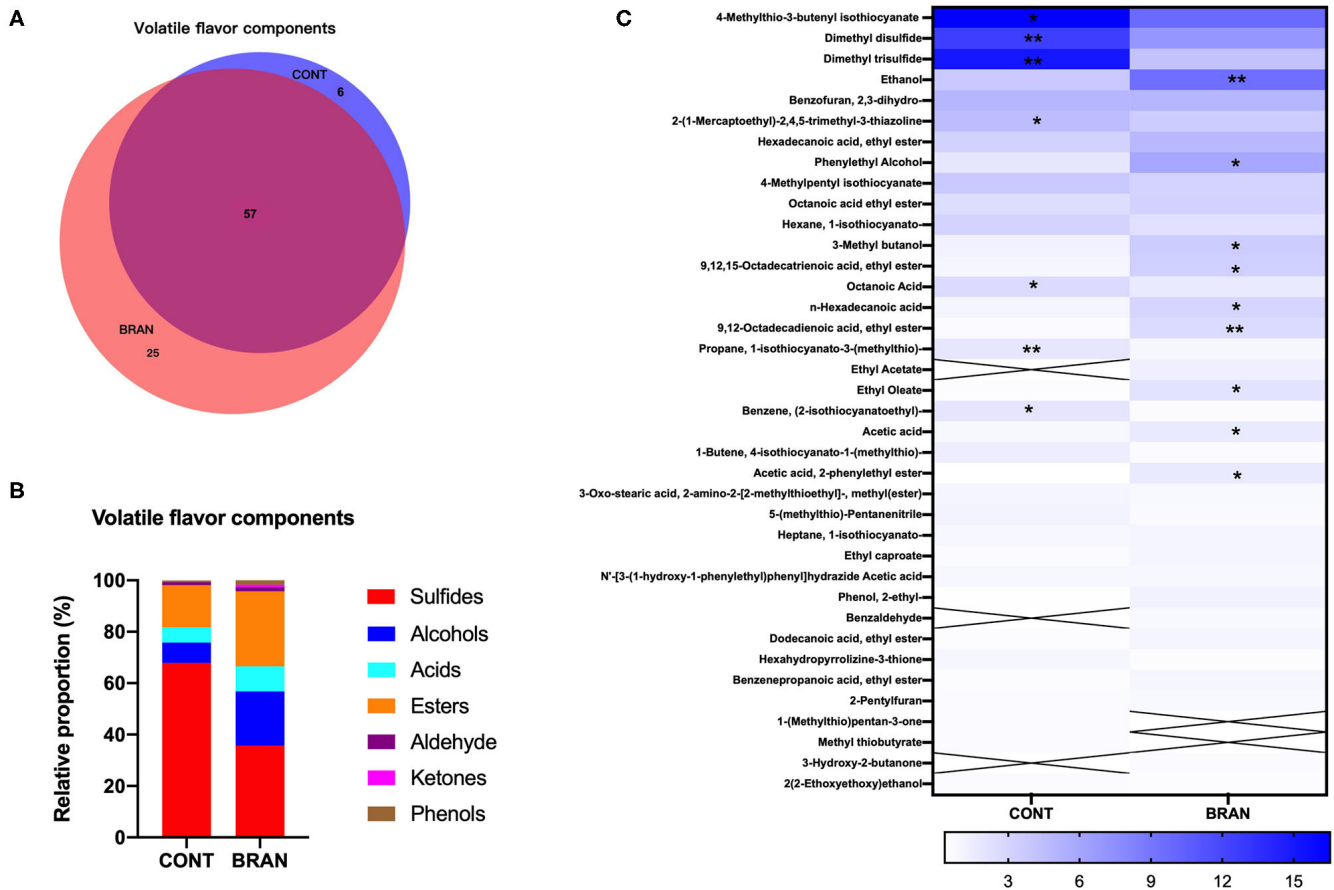
Effect of wheat bran on VF compounds of the ripe pickled radishes on day 28, showing the VEEN analysis of the shared volatiles in the CONT and BRAN groups **(A)**, relative proportions of volatile categories in the CONT and BRAN groups **(B)**, and heatmap of relative volatile abundance (> 0.5%) of the ripe pickled radishes on day 28 **(C)**. Significant differences within each volatile value between CONT and BRAN are marked as *p < 0.05, **p < 0.01.

A total of 88 volatile flavors (VFs) were detected in the ripe pickled radishes on the 28th day (Figure 4), and Supplementary Table S1 lists the detailed information. Wheat bran supplementation resulted in the detection of more VFs in the BRAN group. VEEN analysis showed that 57 out of 88 VFs were shared by both groups, compared to 25 and 6 unique VFs in the BRAN and COS groups, respectively (Figure 4A). The VF compounds were divided into seven categories, namely sulfur-containing compounds (sulfides), alcohols, acids, esters, aldehyde, ketones, and phenols (Figure 4B). Sulfides were the most abundant, followed by esters and alcohols in the ripe radish pickles. Specifically, 22 sulfides, nine alcohols, eight acids, 18 esters, three aldehydes, and three phenols were detected in the CONT group, while 20 sulfides, 13 alcohols, 10 acids, 27 esters, five aldehydes, three ketones, and three phenols were found in the BRAN group. Wheat bran addition resulted in a relatively lower abundance of sulfides (35.63 vs. 67.85%, *p* < 0.001), and a higher abundance of alcohols (21.16 vs. 7.92%, *p* = 0.004), acids (9.79 vs 6.04%, *p* < 0.001), esters (29.09 vs. 16.31%, *p* < 0.001), ketones (0.94 vs. 0.00%, *p* < 0.001).

VFs found with relative abundance (> 0.5%) are presented in Figure 4C. Wheat bran addition resulted in the increased abundance of (A1) ethanol (9.47 vs. 3.73%, *p* = 0.008), (A12) phenylethyl alcohol (5.86 vs 1.84%, *p* = 0.020), (E22) octadecatrienoic acid ethyl ester (1.63 vs. 0.81%, *p* = 0.020), (C2) acetic acid (3.34 vs. 1.84%, *p* = 0.034), and a decreased abundance of (S9) 4-Methylthio-3-butenyl isothiocyanate (4-MTBI) (9.79 vs. 16.52%, *p* = 0.032), (S21) dimethyl disulfide 6.95 vs 12.54%, *p* = 0.003), and (S23) dimethyl trisulfide (4.19 vs. 15.01%, *p* = 0.007).

### Comparison of Bacterial Communities Between the CONT and BRAN Groups

An overview of the bacterial community dynamics during the entire fermentation process of radish pickling was described in our previous paper (not yet published). In this study, we focused on the bacterial communities of epidermal adhesive bacteria and endophytes in ripe pickled radishes on the 28th day. We found that wheat bran supplementation resulted in a reduced number of bacterial OTUs in the BRAN group compared to the CONT group. VEEN analysis also showed that 149 out of 263 OTUs were shared by both groups, compared to 88 and 26 unique OTUs in the CONT and BRAN groups, respectively (Supplementary Figure S2). Differences in the bacterial community profiles between the two groups were investigated by principal component analysis (PCA). As shown in Supplementary Figure S3, the PCA score plot failed to separate the wheat bran-treated samples from the control samples (R = 0.111, *p* = 0.311). Collectively, the data indicated that wheat bran did not significantly affect bacterial alpha and beta diversity in the ripe pickled radishes, may due to a number of insufficient biological samples.

**Figure 5:**
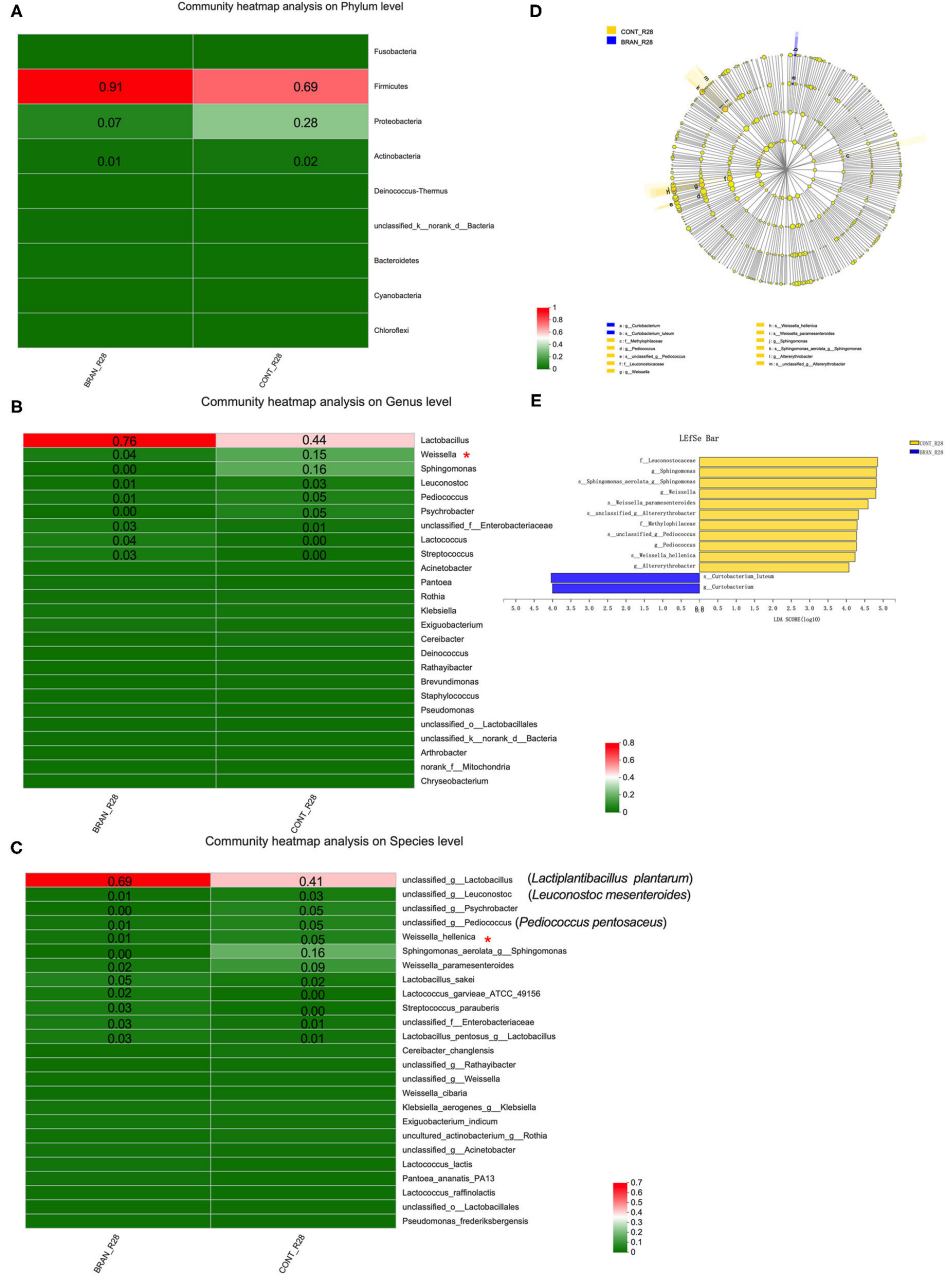
Effect of wheat bran on microbial composition, showing the heat maps of the mean relative abundance of the prominent phyla **(A)**, genera **(B)**, and species **(C)** in the ripe pickled radishes on the 28th day (relative abundance > 0.01 was marked). A Wilcoxon rank-sum test was used to compare bacterial quantity at the phylum and genus levels between the CONT and BRAN groups, and significant differences are marked by *p < 0.05. The LEfSe analysis of microbial abundance from order to species level is shown in **(D)**, and LDA score assessments of the size differentiations between the CONT and BRAN groups, with a score threshold of 4.0, are shown in **(E)**.

**Figure 6:**
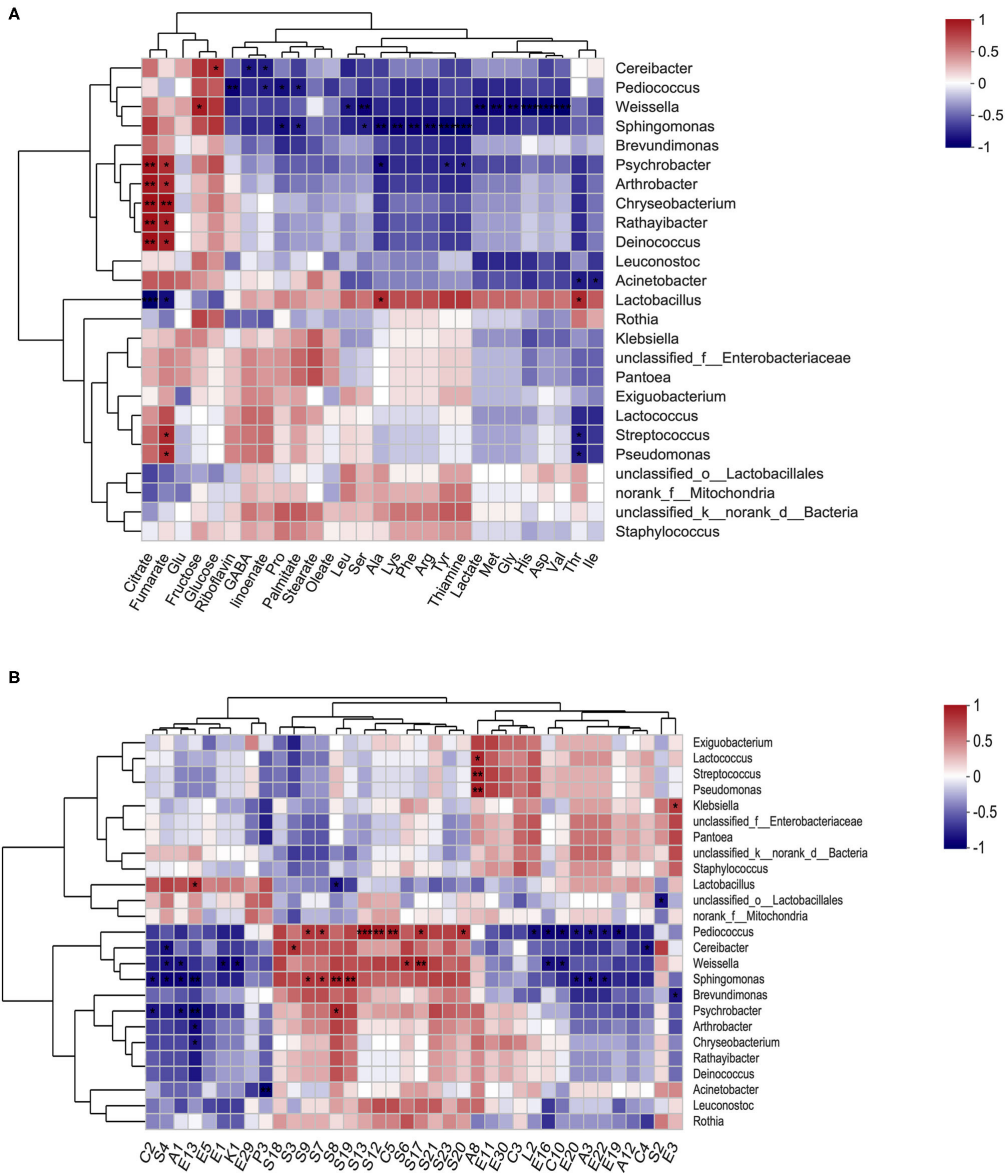
Correlation between microbial structure and metabolite indices. A heatmap of Spearman's correlation between the prominent genera (top 25) and nutrients **(A)**, and the prominent genera (top 25) and volatiles (with relative abundance > 0.5%) **(B)**. The color intensity represents the degree of association (red, positive correlation; blue, negative correlation), and significant correlations are marked by *p < 0.05, **p < 0.01, and ***p < 0.001.

Figures 5A–C show the bacterial communities at the phylum, genus, and species levels, respectively. Firmicutes and Proteobacteria were the prominent phyla species identified at the mature stage (day 28) of pickled radishes (Figure 5A). Wheat bran addition led to an increased amount of Firmicutes (0.91 vs. 0.69) and decreased amount of Proteobacteria (0.07 vs. 0.28). At the genus level, *Lactobacillus, Lactococcus*, and *Streptococcus* were more abundant in the BRAN group (0.76, 0.04, and 0.03, respectively) than in the CONT group (0.44, 0.00, and 0.00, respectively). However, the quantities of *Weissella, Sphingomonas, Leuconostoc, Pediococcus*, and *Psychrobacter* were reduced from 0.15 to 0.04, 0.16 to 0.00, 0.03 to 0.01, 0.05 to 0.01, and 0.05 to 0.00, respectively, after wheat bran treatment (Figure 5B). Only the *Weissella* genus and *W. hellenica* species showed significant differences (Figures 5C,D). According to the results from our culture-dependent method, *Lactiplantibacillus* (*Lp*.) *plantarum* (formerly *Lactobacillus plantarum*) was the most abundant species in the CONT and BRAN groups (Figure 6C).

The linear discriminant analysis (LDA) effect size (LEfSe) method was used to identify the bacterial taxa with significant quantity differences between the CONT and BRAN groups. As shown in Figures 5D, 13 bacterial clades presented statistically significant differences results, with an LDA score of 4.0 (Figure 5E). According to the Wilcoxon rank sum test, wheat bran suppressed the growth of the *Weissella* genus and *W. hellenica* species bacteria. Furthermore, BRAN samples exhibited greater proportions of *Curtobacterium* genus and *C. luteum* species, whereas the CONT samples had greater relative proportions of *Sphingomonas, Pediococcus*, and *Altererythrobacter* bacteria.

### The Relationship Between the Metabolites and the Bacterial Communities

The relationship between the various nutrients (FAAs, GABA, organic acids, fatty acids, sugars, and B vitamins), main volatiles compounds (sulfides, alcohols, acids, and esters), and representative bacterial genus (Figure 6), and species (Supplementary Figure S4) were explored using Spearman's correlations and distance-based redundancy analysis (db-RDA) (Supplementary Figure S5). The results showed that among the organic acids, citrate and fumarate were positively correlated with *Psychrobacter, Arthrobacter, Chryseobacterium, Rathayibacter*, and *Deinococcus* bacteria, but were negatively correlated with *Lactobacillus*. Unlike organic acids (citrate and fumarate), sugars (fructose and glucose), glutamine and riboflavin, and most nutrients (GABA, fatty acids, lactate, thiamine, and most of the FAAs) showed a potential positive correlation with *Lactobacillus*, but a potential negative correlation with *Cereibacter, Pediococcus, Weissella, Sphingomonas*, and *Psychrobacter*. However, only two sweet-tasting FFAs, Ala and Thr, were significantly (*p* < 0.05) correlated with the presence of *Lactobacillus*. *Cereibacter* exhibited a significant negative correlation with the contents of GABA and a-linoenate (*p* < 0.05) and a significant positive correlation with the amount of glucose (*p* < 0.05). *Pediococcus* exhibited a significant negative correlation with the amount of riboflavin, a-linoenate, Pro, and palmitate (*p* < 0.05). In addition, *Weissella* exhibited a significantly negative correlation with Leu, Ser, lactate, Met, Gly, His, Asp, and Val (*p* < 0.05) content. Furthermore, potentially pathogenic strains such as *Sphingomonas* and *Psychrobacter* were significantly negatively correlated with the presence of palmitate, Pro, Ala, Lys, Phe, Arg, Tyr, and thiaminate, or Ala, Tyr, and thiaminate, respectively (*p* < 0.05).

Among the 38 volatile compounds, most sulfides, and the only acid (C5, octanoic acid) showed a potentially positive correlation with *Pediococcus, Cereibacter, Weissella, Sphingomonas, Brevundimonas*, and *Psychrobacter* bacteria, but potentially negative correlated with *Lactobacillus*. However, reverse trends were found for most acids, specifically esters, alcohols, ketones, and the only sulfide (S4, Heptane, 1-isothiocyanato). Furthermore, a significant positive correlation with *Lactobacillus* was only found in E13 (Benzenepropanoic acid, ethyl ester), and a negative correlation was found in S8 (Benzene, 2-isothiocyanatoethyl) (*p* < 0.05). Specifically, S9 (4-MTBI) was positively correlated with *Sphingomonas aerolata* (*p* < 0.001) and *Pediococcus pentosansaceus* (*p* < 0.05) (Supplementary Figure S4B).

A similar relationship was found by db-RDA, which showed that the enriched *Lactobacillus* in the BRAN group was positively correlated with concentrations of lactate, thiamine, GABA, and a-linoenate (Supplementary Figure S5A). For the volatile compounds, *Lactobacillus* in the BRAN group was positively correlated with concentrations of A1 (ethanol) and C2 (acetic acid), while *Pediococcus, Weissella, Sphingomonas*, and *Psychrobacter*, which were enriched in the CONT group, were positively correlated with S9 (4-MTBI), S21 (dimethyl disulfide), and S23 (dimethyl trisulfide) concentrations (Supplementary Figure S5B).

## Discussion

Fermented vegetables are currently attracting increasing attention due to their pleasant taste, high nutritional, and functional values with appreciated health benefits, and can act as suitable probiotic delivery vehicles (30). Salt pickled radish root is a very popular vegetable dish worldwide, especially in Japan, China, and Korea (1, 31, 32). Unlike Japanese *takuan-zuke*, which uses salt and/or rice bran maturation, we utilized wheat bran for nutrient fortification purposes. Our study compared the differences in the nutritional values (FAAs, GABA, organic acids, fatty acids, sugar, and B vitamins), flavor profiles, and microbial properties of pickled radishes treated with or without wheat bran during fermentation, and we revealed their correlations.

Wheat bran is a highly nutritional by-product that is separated during the milling process, making it economical and readily available. However, bioactive compounds such as dietary fibers, proteins, amino acids, minerals, B vitamins, and bioactive compounds are trapped in the cell walls, and thus exhibit low bio-accessibility (33, 34). However, the fermentation processes can target the structure of bran, which has been studied due to its enhanced nutritional potential (35). Studies have shown that the incorporation of wheat bran in proper proportions adds texture, nutrition, and a full-bodied taste to some products (34, 36). In this study, we found that wheat bran supplementation increased the content of most of the tested nutrients in the pickled radishes, except levels of glutamate, citrate, fumarate, fructose, and glucose. The nutritional enhancement effect of wheat bran was expected and was in line with other studies that found that the addition of rice bran increased the levels of niacin, glutamate, and acetate of the fermented radishes (4).

Glutamate is a precursor for proline and GABA synthesis (4). Unlike Rao et al. (6), who reported that threonine is the most abundant FAA in radishes fermented with aged brine during all stages of the fermentation process, we found that glutamate was the most abundant FAA during the maturation stage, followed by threonine. Similarly, glutamate is also the most abundant FAA in Korean kimchi, which is also prepared with radishes (37). The accumulation of glutamate in the fermented radishes may be the result of sodium glutamate, which is added as a condiment during fermentation. Interestingly, more glutamate was consumed during the radish fermentation process with wheat bran. Thus, considering the higher concentrations of proline, arginine, and GABA detected in the BRAN group compared to the CONT group, the increase in glutamate consumption was likely related to the promotion of secondary metabolism of the GABA pathway.

GABA is a non-protein amino acid that is widely found in microorganisms, animals, various vegetables, fruits, and fermented foods, and is produced via the enzymatic decarboxylation of L-glutamate. The physiological functions of GABA as a neurotransmitter on human health include alleviating hypertension, neurological, and sleep disorders, as well as diuretic effects (38, 39). The dehydration process for *takuan-zuke* has been shown to increase the concentration of GABA (40), which was consistent with our findings. This is because the dehydration treatment imposed by salt and weight are strong stressors for radish, which facilitates activation of GABA synthesis while inhibiting GABA metabolism. Initially, GABA levels in brine were expected to be lower than those in radishes. Therefore, GABA permeated from the radish into the brine to reach equilibrium depending on the concentration gradient. Later, with the continuous conversion of GABA from glutamate, the concentrations of GABA in both radish and brine began to increase again. This explains our observation that GABA levels first decreased duration maturation and then increased gain in pickled radishes. However, since we did not analyze the glutamate decarboxylase activities as well as GABA levels the brine, these conclusions may be tentative. We found that fermentation with wheat bran yielded higher GABA, which was consistent with the results of Oda et al. (41) using rice bran bed fermentation. Both the transformation of GABA by plant endogenous enzymes triggered by environmental stresses, or the production of GABA-producing microorganisms such as *Lactobacillus*, may contribute to the observed GABA content in the pickled radishes (39). However, the question of relative contributions of these two pathways to GABA content in pickled radishes remains unanswered. In our previous study, no variations in NaCl content were found between the CONT and BRAN groups, indicating that salt-induced intense osmotic stress was similar between these two groups. Thus, the increased GABA content in the wheat bran fermented radishes was possibly due to the enrichment of GABA-producing microorganisms such as *Lp. plantarum* (42). Of the various investigated fatty acids, α-linolenate, which is an N-3 type polyunsaturated fatty acids, is known to lower systolic blood pressure and to have antimutagenic properties (4). Thiamine and riboflavin, the most commonly investigated B vitamins, are water-soluble molecules that play various roles in cellular functionality, acting as coenzymes in many anabolic and catabolic enzymatic reactions (43). In this study, levels of α-linolenate, thiamine, and riboflavin all increased in the pickled radishes as a result of wheat bran addition. Therefore, radish fermentation with wheat bran will increase its functional value in terms of its health benefits.

Glucose and fructose, which constitute a major component of radishes, will also make them susceptible to dehydration, salting, and affect the organoleptic quality of the pickled products (4). Reducing sugar is also the main carbon source for microorganisms in fermented vegetables. Thus, the content of reducing sugar can indicate the metabolic activity of microorganisms in fermented vegetables (44). Glucose and fructose are utilized by LAB through glycolysis, producing pyruvate, which is the central compound for lactic acid, acetic acid, alcohol, and ester formation (9). Glucose and fructose concentrations were highest in the fresh radish roots; however, the levels were reduced due to dehydration in the BRAN group. After wheat bran treatment, both fructose and glucose levels in the BRAN group significantly declined, compared to the control, indicating that wheat bran treatment was directly impacted reducing sugar content and bacterial metabolic activity. The reduced glucose and fructose content of BRAN samples possibly verified the notion that an increase in reducing sugar consumption is related to the increase LAB metabolic activities. Thus, increasing proportions of lactic acid, acetic acid, ethanol, and ester compounds in the BRAN samples also indirectly supported this assumption.

In general, flavors can be simplify categorized into two groups: non-volatile and volatile compounds. Non-volatile components, such as umami components including amino acids and organic acids, are important contributors to fermented vegetables. In addition, isothiocyanates, alcohols, acetic acid, carbonyl compounds, and acetals are the important volatile flavor components of pickled radishes (6, 8). In this study, the quantity of volatile sulfur compounds significantly decreased, while the proportion of alcohol, ester, acid, and ketone compounds significantly increased during radish fermentation in the presence of wheat bran. Thus, the volatiles in the control pickled radishes were dominated and characterized by sulfides, especially 4-MTBI, dimethyl trisulfide, and dimethyl disulfide. In the presence of wheat bran, 4-MTBI and ethanol were the dominant volatiles in the pickled radishes. 4-MTBI, which is released from 4-methylthio-3-butenyl glucosinolate by endogenous myrosinase hydrolysis, is mainly considered as the principal isothiocyanate generating pungency in radish roots and contributed to the yellow coloring observed during fermentation. Numerous studies have shown that isothiocyanates can act as cancer chemopreventive agents (45–47). However, volatile sulfur compounds have an extremely low odor threshold, and high concentrations of sulfides may cause off-flavors (48). Furthermore, dimethyl disulfide and dimethyl trisulfide are related to the microbial degradation of the sulfur-containing amino acids cysteine and methionine (49). They have a garlic-like odor and onion-like flavor, and thus are not only malodorous but also toxic for all organisms (50). Therefore, when fermented together with wheat bran, it was odor-friendly in the radish roots and can enrich the odor richness and reduce possible undesirable odors.

Microorganisms, that can influence the sensory, nutritional, and functional properties of fermented foods through a variety of metabolic pathways, play a key role in radish fermentation. Thus, the bacterial community profile of epidermal adhesive bacteria and endophytes of pickled radishes was further investigated using high-throughput sequencing technology and a culture-dependent method (data not shown). The microbes in the brine from Sichuan radish pickles have been mainly characterized as *Lactobacillus, Serratia, Enterobacter*, and *Pediococcus* (51). However, in this study, *Lactobacillus, Weissella, Pediococcus*, and *Leuconostoc* were the main bacteria found in the fermented radishes, and *Lp*. *plantarum* was the predominant species in the ripe pickled radishes. Our findings were in accordance with previous studies, which also show that *Lp*. *plantarum* was the most common bacterial species in fermented vegetables (52, 53). Nevertheless, 16S rRNA gene sequencing (DNA-based) may overestimate the abundance of certain species for its inability to differentiate between live and dead bacteria in the brine. Therefore, we suggested that the autochthonous microbiota of the radish root itself, rather than microbiota in the brine, should be monitored. Thus, due to an insufficient number of biological samples, the significant effects of wheat bran on the alpha and beta diversity of ripe pickled radishes were not noticeable on day 28. However, overall, wheat bran supported the growth of *Lactobacillus*, leading to higher lactic acid production, which, in turn, resulted in the fast acidification of the fermentation environment and limited bacterial diversity by suppressing potential pathogenic and spoilage bacteria such as *Sphingomonas* and *Psychrobacter*.

Numerous studies have found a correlation between microbiota and flavor compounds in fermented pickles (6, 12, 24, 28, 54). Remarkably, most associations were revealed based on Spearman's rank correlations, but they did not imply that those specific metabolites were directly produced by the taxa, which could possibly explain the different results found among the different studies. Furthermore, the utilization of sugar substrates by hetero-fermentative and homo-fermentative LABs such as *Lactobacillus, Leuconostoc, Lactococcus, Weissella, Pediococcus* to produce lactic acid, ethanol, and acetic acid was found to be common in all vegetable fermentation processes (55). Meanwhile, these LABs also secreted peptidase to decompose proteins into a variety of amino acids. Amino and organic acids are the main non-volatile components in fermented vegetables. Thus, it was not surprising that levels of lactic acid, acetic acid, ethanol, and most FAAs were positively correlated with the presence of *Lactobacillus*. Furthermore, these primary metabolites were decomposed into secondary volatile metabolites. For instance, alcohols and organic acids may be used to synthesize esters via esterification reactions by the action of microorganisms. Aldehydes and ketones may also be generated via the degradation of branched-chain amino acids by microbial transaminases (56). In addition, LABs may also indirectly affect the flavor of fermented vegetables by reducing the amount of undesirable sulfur compounds due to the inhibition of the enzyme(s) or microbe(s) that produce these sulfides (48). Our results indicated that by stimulating the growth of *Lactobacillus*, wheat bran addition contributed to the production of complex and desirable flavor components, which together improved the flavor, taste, and other properties of the pickled radishes.

## Conclusions

To the best of our knowledge, our study is the first to provide an overview of effect of wheat bran on nutritional content dynamics, flavor components, and microbial community of fermented pickled radish. Wheat bran provided additional nutritional and functional value to the radishes by increasing the amount of most FAAs, and the level of potential functional ingredients such as GABA, α-linolenate, thiamine and riboflavin. Moreover, wheat bran increased the amount of alcohol, ester, acid, and ketones compounds but reduced the number of sulfides (such as dimethyl disulfide and dimethyl trisulfide), which increased the aroma but reduced the off flavors. The enrichment of nutrients and aroma of pickled radish co-fermented with wheat barn was apparently associated with *Lactobacillus*, which was boosted by wheat bran supplementation. These results not only expanded our understanding on the factors that influenced the picked radish quality, but also provide a new strategy to improving the nutritional and flavor properties of fermented vegetables. Future research should explore the co-fermentation of other vegetables with wheat bran, and the potential of this processing technique to provide consumers with more palatable and healthier products.

## Data Availability Statement

The obtained sequence data were deposited in the NCBI Sequence Read Archive (SRA) database under the BioProject ID PRJNA631582. Other raw data supporting the conclusions of this paper will be provided by the authors.

## Author Contributions

XL: conceptualization, investigation, writing—original draft, review and editing, methodology, and funding acquisition. DL: project administration, funding acquisition, sources, and writing—review and editing. All authors contributed to the article and approved the submitted version.

## Funding

This work was funded by the young talent training project of Zhejiang Academy of Agricultural Sciences (2019R15R08E03). The funder had no influence on study design, the collection, analysis and interpretation of data, the writing of the manuscript, and the decision to submit the manuscript for publication.

## Conflict of Interest

The authors declare that the research was conducted in the absence of any commercial or financial relationships that could be construed as a potential conflictof interest.

## Publisher's Note

All claims expressed in this article are solely those of the authors and do not necessarily represent those of their affiliated organizations, or those of the publisher, the editors and the reviewers. Any product that may be evaluated in this article, or claim that may be made by its manufacturer, is not guaranteed or endorsed by the publisher.
