# Supplementary material for: Nutritional Content Dynamics and Correlation of Bacterial Communities and Metabolites in Fermented Pickled Radishes Supplemented With Wheat Bran
Source: Front Nutr. 2022 Mar 8;9:840641. doi: 10.3389/fnut.2022.840641 (PMC8957936; doi:10.3389/fnut.2022.840641)
Supplement: Supplementary file 1 [file Data_Sheet_1.docx]

**Nutritional content dynamics and correlation of bacterial communities and metabolites in fermented pickled radishes supplemented with wheat bran**

Authors: Xiaoqiong Li, Daqun Liu*

Food Science Institute, Zhejiang Academy of Agricultural Sciences, Hangzhou 310021, China

*Corresponding: Daqun Liu

Food Science Institute, Zhejiang Academy of Agricultural Sciences, Hangzhou 310021, China

Email: [daqun.liu@hotmail.com](mailto:daqun.liu@hotmail.com); Fax: +86 (057186722745)

Table S1. The relative abundance of volitive flavor compounds detected in ripe pickled radishes with and without wheat bran

|  | |  | | |  | | CONT | | | | BRAN | | | |
| --- | --- | --- | --- | --- | --- | --- | --- | --- | --- | --- | --- | --- | --- | --- |
| Group (No.) | RT (min) | | CAS No. | Volatile compound | | Chemical formula | 1 | 2 | 3 | Mean | 1 | 2 | 3 | Mean |
| Sulfides |  | |  |  | |  |  |  |  |  |  |  |  |  |
| S1 | 15.77 | | 629-12-9 | n-Pentyl isothiocyanate | | C6H11NS | 0.50 | 0.36 | 0.23 | 0.36 | 0.13 | 0.13 | 0.18 | 0.15 |
| S2 | 17.38 | | 17608-07-0 | 4-Methylpentyl isothiocyanate | | C7H13NS | 4.65 | 3.92 | 2.57 | 3.71 | 3.15 | 3.10 | 2.83 | 3.03 |
| S3 | 18.55 | | 4404-45-9 | Hexane, 1-isothiocyanato- | | C7H13NS | 3.38 | 3.39 | 2.26 | 3.01 | 2.25 | 1.97 | 2.29 | 2.17 |
| S4 | 19.61 | | 4426-83-9 | Heptane, 1-isothiocyanato- | | C8H15NS | 0.72 | nd | 0.82 | 0.77 | 0.85 | 0.90 | 0.96 | 0.90 |
| S5 | 19.88 | | 206761-72-0 | 3-Methylhexyl isothiocyanate | | C8H15NS | 0.39 | 0.53 | 0.30 | 0.41 | 0.27 | 0.22 | 0.19 | 0.23 |
| S6 | 24.30 | | 505-79-3 | Propane, 1-isothiocyanato-3-(methylthio)- | | C5H9NS2 | 1.94 | 1.81 | 1.70 | 1.82 | 0.90 | 0.92 | 0.86 | 0.89 |
| S7 | 26.64 | | 51598-96-0 | 1-Butene, 4-isothiocyanato-1-(methylthio)- | | C6H9NS2 | 0.81 | 1.88 | 1.65 | 1.45 | 0.41 | 0.59 | 0.67 | 0.56 |
| S8 | 26.79 | | 2257/9/2 | Benzene, (2-isothiocyanatoethyl)- | | C9H9NS | 1.40 | 2.48 | 1.86 | 1.92 | 0.68 | 0.67 | 0.35 | 0.57 |
| S9 | 25.92 | | 13028-50-7 | 4-Methylthio-3-butenyl isothiocyanate | | C6H9NS2 | 15.64 | 17.65 | 16.28 | 16.52 | 8.29 | 8.30 | 12.76 | 9.79 |
| S10 | 16.96 | | 5271-38-5 | 2-(methylthio)-Ethanol | | C3H8OS | nd | nd | nd | nd | 0.17 | 0.23 | 0.27 | 0.22 |
| S11 | 1.72 | | 74-93-1 | Methanethiol | | CH4S | 0.54 | 0.45 | 0.51 | 0.50 | 0.22 | 0.23 | 0.17 | 0.21 |
| S12 | 24.77 | | 84310-28-1 | 2-(1-Mercaptoethyl)-2,4,5-trimethyl-3-thiazoline | | C8H15NS2 | 4.47 | 4.26 | 4.98 | 4.57 | 3.29 | 3.80 | 3.57 | 3.55 |
| S13 | 28.19 | | 161364-06-3 | 3-Oxo-stearic acid | | C22H43NO3S | 1.00 | 0.88 | 1.19 | 1.02 | 0.68 | 0.76 | 0.84 | 0.76 |
| S14 | 13.41 | | 2432-79-3 | Hexanoic acid thio S-butyl ester | | C10H20OS | 0.33 | 0.18 | 0.19 | 0.23 | 0.11 | 0.20 | nd | 0.15 |
| S15 | 27.2 | | 4430-42-6 | 5-Methylthiopentyl isothiocyanate | | C7H13NS2 | 0.47 | 0.48 | 1.02 | 0.66 | 0.18 | 0.09 | 0.23 | 0.17 |
| S16 | 4.82 | | 153-08-3 | **Methylthiolacetate** | | C3H6OS | 0.25 | nd | 0.19 | 0.22 | nd | nd | nd | nd |
| S17 | 8.11 | | 2432-51-1 | **Methyl thiobutyrate** | | C5H10OS | 0.93 | 0.35 | 0.51 | 0.60 | nd | nd | nd | nd |
| S18 | 19.83 | | N/A | Hexahydropyrrolizine-3-thione | | C7H11NS | 1.17 | 0.88 | 0.58 | 0.88 | 0.46 | 0.45 | 0.50 | 0.47 |
| S19 | 18.66 | | 66735-69-1 | **1-(Methylthio)pentan-3-one** | | C6H12OS | 0.42 | 0.74 | 0.71 | 0.62 | nd | nd | nd | nd |
| S20 | 23.68 | | 59121-25-4 | 5-(methylthio)-Pentanenitrile | | C6H11NS | 0.88 | 1.04 | 1.31 | 1.08 | 0.53 | 0.78 | 0.66 | 0.66 |
| S21 | 5.18 | | 624-92-0 | Dimethyl disulfide | | C2H6S2 | 11.71 | 12.57 | 13.33 | 12.54 | 6.90 | 8.06 | 5.88 | 6.95 |
| S22 | 10.09 | | 7133-37-1 | Cyclohexyl methyl sulfide | | C7H14S | 0.29 | 0.36 | 0.25 | 0.30 | 0.10 | 0.12 | nd | 0.11 |
| S23 | 12.72 | | 3658-80-8 | Dimethyl trisulfide | | C2H6S3 | 12.56 | 16.70 | 15.76 | 15.01 | 3.49 | 5.04 | 4.02 | 4.19 |
| Alcohols |  | |  |  | |  |  |  |  |  |  |  |  |  |
| A1 | 3.11 | | 64-17-5 | Ethanol | | C2H6O | 5.49 | 2.91 | 2.78 | 3.73 | 8.60 | 9.03 | 10.76 | 9.46 |
| A2 | 5.66 | | 78-83-1 | 1-Propanol | | C4H10O | nd | nd | nd | nd | 0.38 | 0.19 | 0.23 | 0.27 |
| A3 | 8.38 | | 123-51-3 | 3-Methyl butanol | | C5H12O | 2.13 | 0.55 | 0.92 | 1.20 | 4.15 | 3.43 | 3.04 | 3.54 |
| A4 | 12.17 | | 111-27-3 | Hexyl alcohol | | C6H14O | 0.26 | 0.18 | 0.18 | 4.21 | 0.28 | 0.46 | 0.27 | 0.34 |
| A5 | 14.77 | | 3391-86-4 | 1-Octen-3-ol | | C8H16O | 0.20 | nd | nd | 0.07 | 0.09 | 0.17 | 0.00 | 0.09 |
| A6 | 15.96 | | 4534-74-1 | 4-Ethylcyclohexanol | | C8H16O | 0.17 | nd | nd | 0.06 | 0.15 | 0.35 | 0.15 | 0.22 |
| A7 | 18.21 | | 513-85-9 | 2,3-Butanediol | | C4H10O2 | nd | nd | nd | nd | 0.16 | 0.10 | 0.17 | 0.14 |
| A8 | 19.09 | | 111-90-0 | 2(2-Ethoxyethoxy) ethanol | | C6H14O3 | 0.41 | 0.43 | 0.87 | 0.57 | 0.62 | 0.51 | 0.35 | 0.49 |
| A9 | 21.93 | | 57074-37-0 | (Z)-4-Decen-1-ol | | C10H20O | nd | nd | nd | nd | 0.26 | 0.13 | 0.46 | 0.28 |
| A10 | 21.99 | | 10340-22-4 | 3-Decen-1-ol, (Z)- | | C10H20O | nd | nd | nd | nd | 0.18 | 0.00 | 0.00 | 0.06 |
| A11 | 23.23 | | N/A | 3-Methylene-bicyclo[3.2.1]oct-6-en-8-ol | | C9H12O | 0.28 | nd | 0.24 | 0.17 | 0.16 | 0.40 | 0.13 | 0.23 |
| A12 | 23.42 | | 0060-12-8 | Phenylethyl Alcohol | | C8H10O | 2.84 | 0.95 | 1.73 | 0.91 | 7.46 | 4.78 | 5.33 | 5.86 |
| A13 | 30.80 | | 2529-64-8 | Estra-1,3,5(10)-trien-17á-ol | | C18H24O | nd | nd | nd | nd | 0.19 | 0.14 | 0.21 | 0.18 |
| Acids |  | |  |  | |  |  |  |  |  |  |  |  |  |
| C1 | 4.56 | | 105-54-4 | Butanoic acid | | C6H12O2 | 0.31 | nd | 0.20 | 0.25 | 0.59 | 0.25 | 0.37 | 0.41 |
| C2 | 14.84 | | 64-19-7 | Acetic acid | | C2H4O2 | 1.09 | 0.56 | 0.77 | 0.81 | 1.71 | 1.25 | 1.93 | 1.63 |
| C3 | 16.69 | | N/A | N'-[3-(1-hydroxy-1-phenylethyl)phenyl]hydrazide Acetic acid | | C16H18N2O2 | 0.83 | 0.87 | 0.69 | 0.79 | 0.88 | 0.91 | 0.54 | 0.78 |
| C4 | 22.25 | | 103-45-7 | Acetic acid, 2-phenylethyl ester | | C10H12O2 | 0.29 | nd | 0.37 | 0.33 | 1.93 | 1.20 | 1.83 | 1.66 |
| C5 | 25.11 | | 124-07-2 | Octanoic Acid | | C8H16O2 | 2.61 | 2.34 | 2.71 | 2.55 | 1.21 | 2.00 | 1.71 | 1.64 |
| C6 | 25.49 | | 3433-16-7 | Nonanoic acid, 9-oxo-,ethyl ester | | C11H20O3 | nd | 0.18 | 0.18 | 0.18 | 0.16 | 0.31 | nd | 0.23 |
| C7 | 26.11 | | 628-97-7 | Hexadecanoic acid, ethyl ester | | C18H36O2 | nd | nd | nd | nd | 0.11 | nd | nd | 0.11 |
| C8 | 26.75 | | 112-39-0 | Hexadecanoic acid, methyl ester | | C17H34O2 | nd | nd | nd | nd | 0.23 | 0.23 | 0.19 | 0.22 |
| C9 | 29.08 | | 143-07-7 | Dodecanoic acid | | C12H24O2 | 0.33 | 0.41 | 0.69 | 0.48 | 0.43 | 0.21 | 0.29 | 0.31 |
| C10 | 29.19 | | 0057-10-3 | n-Hexadecanoic acid | | C16H32O2 | 0.18 | 1.38 | 1.17 | 0.91 | 2.78 | 3.58 | 2.53 | 2.96 |
| Esters |  | |  |  | |  |  |  |  |  |  |  |  |  |
| E1 | 2.48 | | 141-78-6 | Ethyl Acetate | | C4H8O2 | nd | nd | nd | nd | 1.40 | 0.86 | 1.77 | 1.34 |
| E2 | 6.24 | | 123-92-2 | 1-Butanol-3-methyl-acetate | | C7H14O2 | nd | nd | nd | nd | 0.63 | 0.35 | 0.50 | 0.49 |
| E3 | 9.05 | | 123-66-0 | Ethyl caproate | | C8H16O2 | 1.35 | 0.22 | 0.22 | 0.59 | 1.04 | 1.02 | 0.99 | 1.02 |
| E4 | 11.71 | | 106-30-9 | Heptanoic acid ethyl ester | | C9H18O2 | nd | nd | nd | nd | 0.18 | 0.25 | 0.21 | 0.21 |
| E5 | 14.39 | | 106-32-1 | Octanoic acid ethyl ester | | C10H20O2 | 3.00 | 2.36 | 1.92 | 2.42 | 3.45 | 2.53 | 3.57 | 3.18 |
| E6 | 4.06 | | 110-19-0 | Acetic acid-2-methylpropyl ester | | C6H12O2 | nd | nd | nd | nd | 0.15 | nd | nd | 0.15 |
| E7 | 15.62 | | 105-86-2 | (E)-3,7-dimethyl-2,6-Octadien-1-ol formate | | C11H18O2 | 0.29 | 0.24 | nd | 0.27 | 0.12 | 0.13 | nd | 0.12 |
| E8 | 17.79 | | 2351-90-8 | 2-Octenoic acid, ethyl ester | | C10H18O2 | nd | nd | nd | nd | 0.10 | nd | nd | 0.10 |
| E9 | 19.96 | | 93-89-0 | Benzoic acid, ethyl ester | | C9H10O2 | 0.24 | 0.24 | 0.28 | 0.25 | 0.64 | 0.70 | 0.40 | 0.58 |
| E10 | 20.04 | | 76649-16-6 | Ethyl trans-4-decenoate | | C12H22O2 | nd | nd | nd | nd | 0.09 | nd | nd | 0.09 |
| E11 | 22.68 | | 106-33-2 | Dodecanoic acid, ethyl ester | | C14H28O2 | 0.42 | 0.49 | 0.58 | 0.49 | 1.06 | 1.18 | 0.47 | 0.90 |
| E12 | 22.96 | | 2566-89-4 | 5,8,11,14-Eicosatetraenoic acid, methyl ester, (all-Z)- | | C21H34O2 | nd | nd | nd | nd | 0.10 | 0.16 | 0.17 | 0.14 |
| E13 | 23.16 | | 2021-28-5 | Benzenepropanoic acid, ethyl ester | | C11H14O2 | 0.56 | 0.31 | 0.55 | 0.47 | 0.56 | 0.80 | 1.24 | 0.87 |
| E14 | 25.06 | | 124-06-1 | Tetradecanoic acid, ethyl ester | | C16H32O2 | nd | nd | nd | nd | 0.30 | 0.23 | 0.30 | 0.28 |
| E15 | 27.02 | | 56687-68-4 | [1,1'-Bicyclopropyl]-2-octanoic acid, 2'-hexyl-, methyl ester | | C21H38O2 | nd | nd | nd | nd | 0.14 | 0.11 | nd | 0.12 |
| E16 | 27.11 | | 628-97-7 | Hexadecanoic acid, ethyl ester | | C18H36O2 | 2.07 | 4.13 | 3.34 | 3.18 | 5.05 | 4.49 | 4.87 | 4.80 |
| E17 | 27.28 | | 56687-68-4 | 2'-Hexyl-1,1'-bicyclopropane-2-octanoic acid methyl ester | | C21H38O2 | nd | nd | nd | nd | 0.10 | nd | 0.16 | 0.13 |
| E18 | 27.35 | | 54546-22-4 | Ethyl 9-hexadecenoate | | C18H34O2 | 0.25 | 0.18 | nd | 0.22 | 0.55 | 0.37 | 0.32 | 0.41 |
| E19 | 29.13 | | 111-62-6 | Ethyl Oleate | | C20H38O2 | 0.82 | 0.41 | 0.27 | 0.50 | 2.85 | 1.73 | 1.92 | 2.17 |
| E20 | 29.53 | | 2089036 | 9,12-Octadecadienoic acid, ethyl ester | | C20H36O2 | 1.08 | 0.41 | 0.44 | 0.64 | 2.94 | 2.62 | 2.26 | 2.61 |
| E21 | 29.79 | | 2566-89-4 | 5,8,11,14-Eicosatetraenoic acid, methyl ester, (all-Z)- | | C21H34O2 | nd | nd | nd | nd | 0.18 | 0.19 | 0.22 | 0.20 |
| E22 | 30.08 | | 1191-41-9 | 9,12,15-Octadecatrienoic acid, ethyl ester, (Z,Z,Z)- | | C20H34O2 | 1.29 | 0.79 | 0.90 | 0.99 | 4.12 | 3.07 | 2.82 | 3.34 |
| E23 | 30.18 | | 18465-99-1 | 9,12,15-Octadecatrienoic acid, 2,3-dihydroxypropyl ester, (Z,Z,Z)- | | C21H36O4 | nd | nd | nd |  | 0.16 | 0.14 | 0.14 | 0.15 |
| E24 | 30.25 | | N/A | 11,13-Dihydroxy-tetradec-5-ynoic acid, methyl ester | | C15H26O4 | nd | 0.70 | 0.62 | 0.66 | nd | 0.11 | 0.28 | 0.20 |
| E25 | 17.88 | | 2499-59-4 | **2-Propenoic acid, octyl ester** | | C11H20O2 | 0.45 | 0.20 | 0.24 | 0.30 | nd | nd | nd | nd |
| E26 | 27.07 | | 2046-21-1 | **Methyl 6-oxoheptanoate** | | C8H14O3 | 0.27 | 0.25 | 0.21 | 0.24 | nd | nd | nd | nd |
| E27 | 30.86 | | 84-78-6 | **1,2-Benzenedicarboxlic acid, butyloctylester** | | C20H30O4 | nd | nd | 0.23 | 0.23 | nd | nd | nd | nd |
| E28 | 10.85 | | 70424-13-4 | cis-2-(2-Pentenyl)furan | | C9H12O | 0.32 | nd | nd | 0.32 | 0.13 | 0.25 | nd | 0.19 |
| E29 | 28.27 | | 496-16-2 | Benzofuran, 2,3-dihydro- | | C8H8O | 3.86 | 4.97 | 5.88 | 4.90 | 3.90 | 6.11 | 5.04 | 5.02 |
| E30 | 8.97 | | 3777-69-3 | 2-Pentylfuran | | C9H14O | nd | 0.60 | 0.50 | 0.55 | 0.70 | 1.12 | 0.49 | 0.77 |
| Aldehyde |  | |  |  | |  |  |  |  |  |  |  |  |  |
| L1 | 15.08 | | N/A | 2-methyl-3-methylene cyclopentanecarboxaldehyde | | C8H12O | nd | nd | nd | nd | 0.10 | 0.18 | nd | 0.14 |
| L2 | 16.75 | | 100-52-7 | Benzaldehyde | | C7H6O | nd | nd | nd | nd | 1.00 | 0.45 | nd | 0.72 |
| L3 | 18.46 | | 31681-26-2 | α-Propyl-2-furanacetaldehyde | | C9H12O2 | 0.41 | 0.34 | 0.35 | 0.36 | 0.19 | 0.54 | nd | 0.37 |
| L4 | 20.62 | | 4748-78-1 | 4-ethyl-Benzaldehyde | | C9H10O | 0.54 | 0.49 | 0.44 | 0.49 | 0.31 | 0.74 | 0.31 | 0.45 |
| L5 | 14.11 | | 2548-87-0 | (E)-2-Octenal | | C8H14O | 0.46 | 0.25 | 0.17 | 0.29 | 0.25 | 0.72 | 0.32 | 0.43 |
| Ketones |  | |  |  | |  |  |  |  |  |  |  |  |  |
| K1 | 10.26 | | 513-86-0 | 3-Hydroxy-2-butanone | | C4H8O2 | nd | nd | nd | nd | 0.49 | 0.46 | 0.72 | 0.56 |
| K2 | 24.81 | | 104-67-6 | 2(3H)-Furanone, 5-heptyldihydro- | | C11H20O2 | nd | nd | nd | nd | 0.30 | 0.37 | 0.36 | 0.34 |
| K3 | 26.22 | | 77761-61-6 | 12-Hydroxy-14-methyl-oxa-cyclotetradec-6-en-2-one | | C14H24O3 | nd | nd | nd | nd | 0.13 | nd | nd | 0.13 |
| Phenols |  | |  |  | |  |  |  |  |  |  |  |  |  |
| P1 | 21.74 | | 527-35-5 | 2,3,5,6-tetramethyl Phenol | | C10H14O | nd | nd | nd | nd | 0.11 | 0.22 | nd | 0.17 |
| P2 | 24.595 | | 3766-81-2 | 2-(1-methylpropyl)-, methylcarbamate Phenol | | C12H17NO2 | 0.17 | nd | nd | 0.17 | 0.15 | 0.20 | nd | 0.17 |
| P3 | 26.26 | | 90-00-6 | Phenol, 2-ethyl- | | C8H10O | 0.30 | 0.32 | 0.43 | 0.35 | 0.28 | 0.93 | 2.18 | 1.13 |
| P4 | 26.48 | | 7786-61-0 | 2-Methoxy-4-vinylphenol | | C9H10O2 | nd | 0.44 | 0.53 | 0.48 | 0.27 | 0.47 | 0.24 | 0.33 |

CONT: fermented radishes without wheat bran on day 28;

BRAN: fermented radish with wheat bran on day 28;

RT: retention time

nd: not detected

N/A: not available

**Supplementary Figure 1.** Flow chart showing the fermented radish roots grouping and timeline of the experimental protocol. DEHY: dehydration stage, including days 0 (start of dehydration) and 14, CONT: seasoning stage (days 28, 42, and 56) of the fermented radish without wheat bran, and BRAN: seasoning stage (days 28, 42, and 56) of the fermented radish with wheat bran.

**Supplementary Figure 2.** VEEN analysis of shared OTUs in the CONT and BRAN groups on day 28.

**Supplementary Figure 3.** Effect of wheat bran on microbial beta-diversity based on PCA analysis. The distance from the sample point to the projection of the metabolite (nutrients (A), volatiles (B)) indices vector indicates the strength of the samples affected by the indices.

**Supplementary Figure 4.** Correlation between microbial structure and metabolites indices, showing a heatmap of Spearman’s correlation between the prominent species and nutrients (A), and the prominent species and volatiles (B). The intensity of the colors represents the degree of association (red, positive correlation; blue, negative correlation). Significant correlations are marked by *p<0.05; **p<0.01; ***p<0.001.

**Supplementary Figure 5.** Correlation between microbial structure and main metabolites indices. Distance-based redundancy analysis (db-RDA) of the prominent genera in response to the main nutrients (A), and the prominent genera in response to the main volatiles (B).
